# Development of a clinical tool to identify patients with early inflammatory arthritis at high risk of employment loss: analysis from the National Early Inflammatory Arthritis Audit

**DOI:** 10.1093/rap/rkaf149

**Published:** 2025-12-23

**Authors:** Ed Alveyn, Katie Bechman, Maryam Adas, Paul Amlani-Hatcher, Mini Dey, Sarah Gallagher, Mark Gibson, Bethan Jones, Daksh Mehta, Sam Norton, Elizabeth Price, Mark Russell, Karen Walker-Bone, Elizabeth MacPhie, James Galloway

**Affiliations:** Department of Inflammation Biology, Centre of Rheumatic Disease, King’s College London, London, UK; Department of Inflammation Biology, Centre of Rheumatic Disease, King’s College London, London, UK; Department of Inflammation Biology, Centre of Rheumatic Disease, King’s College London, London, UK; NEIAA Patient Panel, British Society for Rheumatology, London, UK; Department of Inflammation Biology, Centre of Rheumatic Disease, King’s College London, London, UK; NEIAA, British Society for Rheumatology, London, UK; Department of Inflammation Biology, Centre of Rheumatic Disease, King’s College London, London, UK; Faculty of Health and Life Sciences, University of Liverpool, Liverpool, UK; Department of Inflammation Biology, Centre of Rheumatic Disease, King’s College London, London, UK; Department of Inflammation Biology, Centre of Rheumatic Disease, King’s College London, London, UK; Department of Psychology, Institute of Psychiatry, Psychology and Neuroscience, King’s College London, London, UK; Department of Rheumatology, Great Western Hospital NHS Foundation Trust, Swindon, UK; Department of Inflammation Biology, Centre of Rheumatic Disease, King’s College London, London, UK; Monash Centre for Occupational and Environmental Health, Monash University, Melbourne, Australia; Rheumatology, Minerva Health Centre, Preston, UK; Department of Inflammation Biology, Centre of Rheumatic Disease, King’s College London, London, UK

**Keywords:** work disability, employment loss, manual occupation, early inflammatory arthritis, risk stratification

## Abstract

**Objectives:**

Work disability is an early consequence of inflammatory arthritis. Preventive interventions exist but access is limited, highlighting the need for risk stratification. We aimed to develop a tool using routinely collected data to identify patients at greatest risk of employment loss.

**Methods:**

This cohort study used data from the National Early Inflammatory Arthritis Audit. Patients ≥16 years with early inflammatory arthritis (EIA), enrolled May 2018–April 2025, employed at diagnosis and with three-month follow-up were included. The outcome was self-reported employment loss at 3 months. Predictors were occupation (manual vs non-manual), age, sex, disease activity (DAS28 > 5.1), mental health (anxiety/depression) and musculoskeletal burden (MSKHQ ≤25v > 25). Employment loss was modelled using Poisson regression. Model discrimination, calibration and bootstrap validation were assessed. A risk score was derived and stratified into low, medium and high-risk groups.

**Results:**

Of 11 894 patients with EIA, 6036 were employed at baseline and 1662 had complete work-outcome data. At 3 months, 168(10.1%) reported employment loss. Manual workers had higher risk than non-manual (14.1% vs 7.8%). In multivariable analysis, manual work (IRR: 1.54, 95% CI: 1.15–2.06), older age (per 10 years: IRR: 1.65, 95% CI: 1.43–1.90), high musculoskeletal burden (IRR: 1.55, 1.10–2.19) and anxiety/depression (IRR: 1.45, 1.02–2.06) were associated with employment loss, whereas DAS28 was not. The optimal model (age, occupation, musculoskeletal and mental health) showed good discrimination (C-statistic 0.710) and calibration. An 8-point score stratified patients into low (2.5%), medium (9.1%) and high-risk (19.5%).

**Conclusion:**

Employment loss in EIA is driven by occupation, age, musculoskeletal symptoms and mental health. A risk tool incorporating these domains can stratify patients and guide targeted interventions.

Key messages

- Among 1600 employed adults with early inflammatory arthritis, one in ten stopped working within 3 months of diagnosis.
- Manual work, older age, female sex, poor mental health and greater MSK burden predicted employment loss.
- A simple 8-point tool stratified risk of early employment loss to support targeted vocational intervention.

## Introduction

Work disability is a major consequence of inflammatory arthritis, with up to 10% of patients losing employment within three months of diagnosis, rising to 15% in manual occupations [1]. Many people stop working very early in their disease course, with the drivers of work disability including individual health and occupational demands [2, 3]. While this highlights the scale and inequity of the problem, clinicians currently lack tools to identify which patients are most likely to require targeted support.

Effective interventions to prevent employment loss exist. Traditionally, government schemes have focused on supporting individuals after they have left employment, with the aim of facilitating return to work; however, new initiatives have been developed to help people remain employed, thereby preventing job loss [4]. Systematic reviews show that integrated approaches combining clinical care, workplace modification, vocational rehabilitation and psychological support can reduce work disability by 30–50% [5–7]. Recent EULAR recommendations further emphasize the need to address work participation proactively [8]. However, resources for occupational health support are scarce, making early risk stratification essential to direct interventions to those most likely to benefit.

Developing effective risk stratification requires comprehensive data on both clinical and occupational factors—information that is rarely available in routine clinical datasets. Many existing studies have been limited by small sample sizes, single-centre recruitment or incomplete occupational characterization, hindering the development of generalizable tools for clinical practice. This gap between the theoretical need for risk assessment and the practical constraints of available data represents a key barrier to implementing systematic approaches to work disability prevention.

In England and Wales, there is a national audit programme (NEIAA) to monitor and drive quality improvement for early inflammatory arthritis care. In NEIAA, data are collected on work using the Work Productivity and Activity Impairment questionnaire, which captures impact but does not quantify future risk to employment. Previous studies have demonstrated the enormous impacts of inflammatory arthritis on work, and we have previously shown that standard quality metrics such as time to referral and speed of initiation of DMARD therapy were not strongly associated with work outcomes, suggesting that we are missing key determinants of employment loss [1].

Risk prediction tools have transformed other areas of rheumatology: the FRAX score enables targeted osteoporosis prevention [9], while cardiovascular calculators such as SCORE guide statin prescribing [10]. A comparable approach for employment loss could allow systematic identification of high-risk patients, enabling more efficient allocation of resources and robust auditing of work outcomes.

This study had two complementary aims: first, to examine predictors of employment loss in a large cohort of newly diagnosed patients with early inflammatory arthritis; and second, to explore the feasibility of developing a pragmatic risk stratification tool using the limited but routinely available clinical data that characterizes real-world rheumatology practice.

## Methods

### Study design and population

We conducted an exploratory clinical prediction model study using the National Early Inflammatory Arthritis Audit (NEIAA), which collects standardized data from all rheumatology units in England and Wales. Eligible participants were adults with early inflammatory arthritis (rheumatoid arthritis, psoriatic arthritis or undifferentiated arthritis) enrolled between May 2018 and April 2025 who were in paid employment at baseline and had 3-month follow-up data on work outcomes.

### Variables and outcome

The primary outcome was employment loss at 3 months (transition from employed to not employed), ascertained by patient self-report.

Occupations were classified by physical demands as manual (moderate/high demands: Standard Occupational Classification [SOC] groups 3: associate professional occupations; 5: skilled trades occupations; 6: caring, leisure and other service occupations; 7: sales and customer service occupations; 8: process, plant and machine operatives and 9: elementary occupations) versus non-manual work (low demands: SOC groups 1: managers, directors and senior officials; 2: professional occupations; 4: administrative and secretarial occupations) [11]. This binary categorization was chosen for pragmatic reasons (i.e. for practical use by clinicians) while also capturing the fundamental distinction between physically demanding and less physically demanding jobs, which are relevant to musculoskeletal conditions.

Clinical variables included age (categorized as <50, 50–59, ≥60 years to avoid conflating retirement with arthritis-related job loss), sex, disease activity (DAS28 categorized above or below 5.1), mental health (probable anxiety/depression using validated PHQ-2/GAD-2 composite score) and musculoskeletal health status (MSKHQ). The MSKHQ is a validated 14-item instrument (range 0–56, lower scores indicating greater impairment) dichotomized at ≤25 vs >25 to capture clinically meaningful differences in work-relevant musculoskeletal function (Supplementary Data S1, available at *Rheumatology Advances in Practice* online).

### Statistical analysis framework

We conducted two complementary analyses with distinct objectives. First, we performed explanatory analyses to identify and quantify factors associated with employment loss, using multivariable regression and causal mediation analysis. Second, we undertook prediction modelling to explore the feasibility of developing a risk stratification tool using routinely available clinical data. Recognizing the constraints of the available predictor set—which omits known important factors such as fatigue, comorbidities, detailed socioeconomic measures and workplace support—we approached this as a proof-of-principle study testing whether useful risk stratification is achievable with pragmatically available data.

### Primary statistical analysis

Employment loss was modelled using Poisson regression with robust standard errors, appropriate for the outcome frequency (10.2% employment loss rate). We report risk ratios (RR) with 95% CIs. Age effects were presented per 10-year increment for clinical interpretability.

### Model development for risk stratification

We adopted a structured approach that combined data-driven variable selection with clinical feasibility considerations. Initially, penalized regression methods (LASSO and elastic net with 10-fold cross-validation) were applied to a comprehensive candidate variable set including: physical job demands, age, sex, baseline disease activity, musculoskeletal health, mental health, socioeconomic deprivation, diagnosis type and detailed occupational categories. This approach identified which variables contributed most to prediction when all available information was considered simultaneously (Supplementary Data S2, available at *Rheumatology Advances in Practice* online).

Subsequently, we evaluated traditional regression models using the variables identified through penalized selection, testing different candidate prediction models with sequential addition of predictor domains. The final model specification balanced statistical performance with practical implementation requirements, retaining variables that were both predictive and consistently available during routine clinical assessment.

### Risk stratification tool development

From the multivariable model, we constructed a pragmatic risk-stratification score designed for use in clinical settings. The tool was limited to routinely available clinical and occupational variables identified from the multivariable regression models as most predictive. Regression coefficients from the final model were used to guide the relative weighting of each predictor, which were then converted into an integer-based scoring system for pragmatic use. To facilitate practical use, total scores were grouped into three risk strata, representing low, medium and high risk of early employment loss. These categories were defined on the basis of both score distribution and the need for clinically meaningful distinctions that could underpin differential care pathways (Supplementary Data S3, available at *Rheumatology Advances in Practice* online).

### Model and stratification tool performance

Discrimination was assessed using the C-statistic. Calibration was evaluated using Hosmer-Lemeshow goodness-of-fit test and calibration plots comparing predicted versus observed risks across deciles. Bootstrap internal validation (200 iterations) assessed optimism and provided bias-corrected performance estimates. Cross-validation (10-fold) provided an additional estimate of model performance on unseen data partitions.

### Mediation analysis

To examine pathways linking occupation to employment loss, we conducted formal causal mediation analysis using the mediate command in Stata. We estimated average treatment effects and decomposed the total effect of occupational demands into natural direct effects (unmediated pathways) and natural indirect effects (mediated through disease activity, mental health and musculoskeletal symptoms) (Supplementary Data S4, available at *Rheumatology Advances in Practice* online).

### Missing data

Of 5287 working patients with occupational data, 68.6% lacked 3-month outcomes. We characterized patterns of missing outcome data by comparing baseline characteristics between those with and without 3-month employment data. Continuous variables were compared using *t*-tests and categorical variables using chi-square tests. We addressed potential biases in missing data using inverse probability weighting and multiple sensitivity analyses under different missing-not-at-random assumptions. Primary analysis used complete cases (*n* = 1662) (Supplementary Data S5, available at *Rheumatology Advances in Practice* online).

### Ethics

NEIAA has ethical approval (REC: 19/EE/0082; CAG: 19/CAG/0059) and is commissioned by the Healthcare Quality Improvement Partnership. Informed patient consent was not required, as NEIAA has permission from the UK Government Secretary of State for Health to collect data for the purposes of national audit.

## Results

### Study population

Of 11 894 patients with early inflammatory arthritis in NEIAA, 6036 (50.7%) were working at baseline. After excluding those with missing occupational data (*n* = 749) and missing 3-month employment outcomes (*n* = 3625), the primary analysis included 1662 patients (Supplementary Fig. S1, available at *Rheumatology Advances in Practice* online). Among patients with complete data at 3 months, 168 (10.1%; 95% CI: 8.7–11.6%) had lost employment.

### Baseline characteristics

The mean age was 50.8 years (s.d. 11.8) and 1116 (67.1%) were female (Table 1). Most patients (1031; 62.0%) worked in non-manual occupations with low physical demands, whilst 631 (38.0%) worked in manual occupations with moderate to high physical demands. The mean baseline DAS28 was 4.58 (s.d. 1.36), with 575 (36.7%) having high disease activity (DAS28 > 5.1). Over half (899; 54.3%) met criteria for probable anxiety or depression based on their PHQ-2/GAD-2 score.

**Table 1.** Baseline characteristics by physical job demands

Characteristic	Total (*N* = 1662)	Non-manual (*N* = 1031)	Manual (*N* = 631)	*P*-value
**Demographics**				
Age, mean (s.d.)	50.8 (11.8)	50.0 (11.5)	52.1 (12.0)	<0.001
Female, *n* (%)	1116 (67.1)	763 (74.0)	353 (55.9)	<0.001
**Clinical characteristics**				
DAS28, mean (s.d.)	4.58 (1.36)	4.49 (1.38)	4.72 (1.31)	0.001
Diagnosis type, *n* (%)				0.087
Rheumatoid arthritis	1222 (73.5)	740 (71.8)	482 (76.4)	
Psoriatic arthritis	277 (16.7)	187 (18.1)	90 (14.3)	
Undifferentiated arthritis	163 (9.8)	104 (10.1)	59 (9.3)	
**Mental health**				
Mental health score, mean (s.d.)	4.5 (3.7)	4.3 (3.5)	4.9 (3.9)	0.001
**MSK Health**				
MSKHQ score, mean (s.d.)	25.9 (11.2)	26.2 (11.0)	25.3 (11.5)	0.111
**Work impairment**				
Overall work impairment %, mean (s.d.)	32.1 (26.4)	30.2 (25.5)	35.4 (27.6)	<0.001
**Socioeconomic characteristics**				
IMD decile, mean (s.d.)	5.8 (2.8)	6.2 (2.7)	5.1 (2.7)	<0.001
Most deprived 30%, *n* (%)	388 (26.2)	197 (21.7)	191 (33.3)	<0.001
**Occupational characteristics**				
Occupation type, *n* (%)				<0.001
Managerial/professional	768 (46.2)	768 (74.5)	0 (0.0)	
Skilled/technical	410 (24.7)	263 (25.5)	147 (23.3)	
Service occupations	303 (18.2)	0 (0.0)	303 (48.0)	
Manual/elementary	181 (10.9)	0 (0.0)	181 (28.7)	

Data presented as mean (s.d.) for continuous variables and *n* (%) for categorical variables. *P*-values from *t*-tests for continuous variables and chi-square tests for categorical variables. Non-manual work includes managers, professionals and administrative roles (SOC groups 1, 2, 4). Manual work includes associate professionals, skilled trades, service workers, sales workers, machine operatives and elementary occupations (SOC groups 3, 5, 6, 7, 8, 9).

IMD, Index of Multiple Deprivation; MSKHQ, MSK Health Questionnaire; SOC, Standard Occupational Classification.

Missing data: DAS28 (*n* = 94), Mental health score (*n* = 7), MSKHQ (*n* = 1), Work impairment (*n* = 136), IMD data (*n* = 180).

Significant differences existed between occupational groups (Table 1). Manual workers were older (mean 52.1 vs 50.0 years, *P* < 0.001), more likely to be male (44.1% vs 26.0% male, *P* < 0.001), had higher disease activity (DAS28 4.72 vs 4.49, *P* = 0.001), worse mental health scores (4.87 vs 4.28, *P* = 0.001) and greater socioeconomic deprivation (33.3% vs 21.7% in most deprived areas, *P* < 0.001).

### Employment outcomes by risk factors

Employment loss rates varied significantly by key risk factors (Table 2). Manual workers had higher employment loss than non-manual workers (14.1% vs 7.8%), with the effect strongest in older age groups. Employment loss increased substantially with age: 6.1% in those <50 years, 7.7% in those 50–59 years and 20.2% in those ≥60 years (*P* < 0.001 for trend).

**Table 2.** Employment loss by occupational and clinical factors

Factor	Category	*N*	Employment loss *n* (%)	95% CI (%)
**Physical demands**				
	Non-manual	1031	80 (7.8)	6.2–9.6
	Manual	631	89 (14.1)	11.6–17.0
**Age groups**				
	<50 years	672	41 (6.1)	4.5–8.2
	50–59 years	574	44 (7.7)	5.8–10.1
	≥60 years	416	84 (20.2)	16.6–24.3
**Probable anxiety or depression**				
	No	756	57 (7.5)	5.9–9.6
	Yes	899	111 (12.3)	10.4–14.7
**Disease impact (MSKHQ)**				
	Better MSK health (>25)	805	61 (7.6)	5.9–9.6
	Worse MSK health (≤25)	856	108 (12.6)	10.6–15.0
**Disease activity**				
	DAS28 ≤ 5.1	993	95 (9.6)	7.9–11.6
	DAS28 > 5.1	575	67 (11.7)	9.3–14.5

Employment loss defined as transition from employed at baseline to not employed at 3-month follow-up. 95% CIs calculated using Wilson method. Manual work includes moderate and high physical demands (SOC groups 3,5,6,7,8,9). Non-manual work includes low physical demands (SOC groups 1,2,4).

DAS28, Disease Activity Score in 28 joints; MSKHQ, MSK Health Questionnaire.

Numbers may not sum to total due to missing data on individual variables.

Mental health status was strongly associated with employment outcomes, with 12.3% of those with probable anxiety or depression losing employment compared with 7.5% of those without anxiety or depression (*P* = 0.004). Similarly, musculoskeletal health status showed clear gradients, with employment loss rates of 7.6% in those with better MSK health (MSKHQ >25) versus 12.6% in those with worse MSK health (MSKHQ ≤25) (*P* = 0.012).

### Missing data patterns

Of the 5287 patients working at baseline who had occupational data, 3625 (68.6%) lacked 3-month employment outcomes. Comparison of baseline characteristics between those with and without outcome data revealed several systematic differences (Supplementary Table S5, available at *Rheumatology Advances in Practice* online). Patients with missing outcomes were younger than complete cases (mean age 49.4 vs 50.9 years, *P* < 0.001) and less likely to be female (62.2% vs 66.9%, *P* < 0.001). The distribution of physical demands differed significantly between groups (*P* = 0.010), with high physical demand occupations more common among those with missing data (17.7% vs 14.8%). Disease activity did not differ significantly between groups (mean DAS28 4.53 vs 4.58, *P* = 0.147).

### Part 1: Explanatory analysis—multivariable model

**Figure 1.**
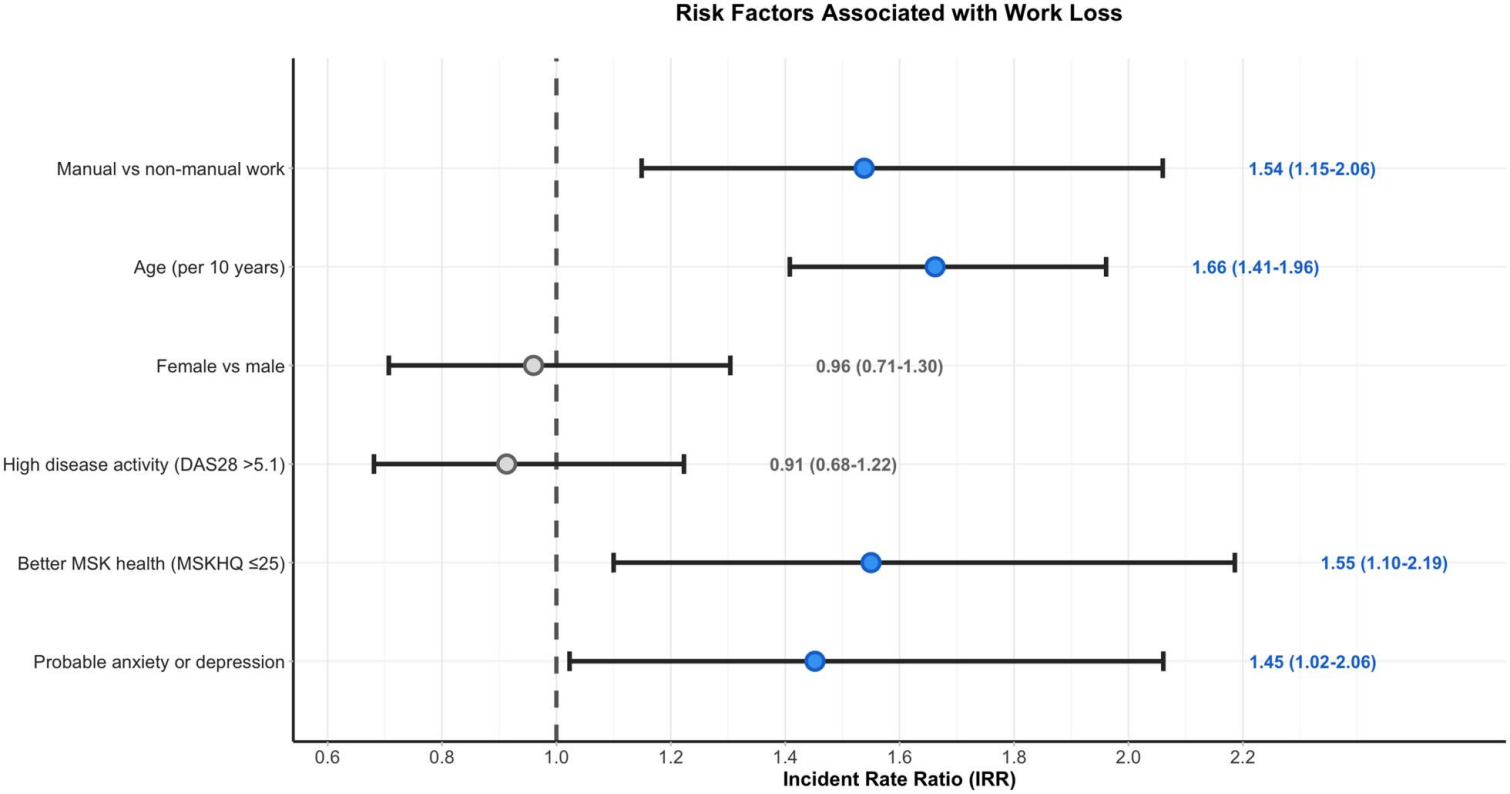
Risk factors for employment loss at 3 months. Forest plot from multivariable Poisson regression. IRR = incidence rate ratio. Reference line at IRR = 1.0

In sequential multivariable models, the association between manual work and employment loss remained robust across all adjustments (Table 3; Fig. 1). In the unadjusted model, manual work was associated with an 82% increased risk of employment loss (RR 1.82, 95% CI: 1.37–2.42). This attenuated but remained significant after adjustment for demographics, disease activity, mental health and musculoskeletal symptom burden (RR 1.54, 95% CI: 1.15–2.06, *P* = 0.004).

**Table 3.** Progressive Poisson regression models—IRR (95% CI)

Variable	Model 1	Model 2	Model 3	Model 4	Model 5
	Occupation only	+ Demographics	+ Disease activity	+ MSK symptoms	+ Mental health
**Manual vs non-manual work**	1.82 (1.37–2.42)	1.65 (1.24–2.21)	1.57 (1.17–2.10)	1.54 (1.15–2.07)	1.54 (1.15–2.06)
**Age (per 10 years)**	–	1.63 (1.40–1.90)	1.61 (1.37–1.89)	1.65 (1.40–1.94)	1.66 (1.41–1.96)
**Female vs male**	–	1.06 (0.78–1.43)	1.01 (0.74–1.37)	0.98 (0.72–1.33)	0.96 (0.71–1.30)
**DAS28 > 5.1 vs ≤5.1**	–	–	1.07 (0.80–1.44)	0.91 (0.68–1.22)	0.91 (0.68–1.22)
**Better MSK health (MSKHQ ≤25)**	–	–	–	1.85 (1.37–2.51)	1.55 (1.10–2.19)
**Probable anxiety or depression**	–	–	–	–	1.45 (1.02–2.06)
***N***	1662	1662	1568	1567	1560

Results from Poisson regression with robust standard errors, reporting incidence rate ratios (IRR) with 95% CIs. Age effects presented per 10-year increment. Manual work includes moderate and high physical demands (SOC groups 3,5,6,7,8,9). Better MSK health defined as MSKHQ ≤25 versus >25. Probable anxiety or depression based on validated PHQ-2/GAD-2 composite score. Sample sizes decrease due to missing covariate data in sequential models.

DAS28, Disease Activity Score in 28 joints; MSKHQ, MSK Health Questionnaire; SOC, Standard Occupational Classification.

Age showed a strong association with employment loss, with each 10-year increase associated with a 65% higher risk in the fully adjusted model (RR 1.65, 95% CI: 1.43–1.90, *P* < 0.001). Worse musculoskeletal health (MSKHQ ≤25) was associated with a 55% increased risk (IRR 1.55, 95% CI: 1.10–2.19, *P* = 0.012), while probable anxiety or depression conferred a 45% increased risk (RR 1.45, 95% CI: 1.02–2.06, *P* = 0.037).

Notably, traditional disease activity measures (DAS28 > 5.1) were not significantly associated with employment loss in adjusted analyses (RR 0.91, 95% CI: 0.68–1.22, *P* = 0.541), suggesting that patient-reported measures may be more relevant for work outcomes than clinical assessments.

### Part 2: Prediction modelling: variable selection and model comparison

Penalized regression approaches initially selected seven variables from the comprehensive candidate set: physical job demands, age, sex, baseline disease activity, musculoskeletal health, mental health and diagnosis type. Both LASSO (λ = 0.0036) and elastic net (α = 0.5, λ = 0.0060) identified identical variables, excluding socioeconomic deprivation and detailed occupational categories.

However, diagnosis type contributed no predictive value when formally tested (Wald test: χ^2^=0.28, *P* = 0.871) and was excluded from the final model specification. Four prediction approaches were compared using the remaining variables: standard logistic regression achieved optimal discrimination (C-statistic 0.708), outperforming LASSO (0.704), elastic net (0.704) and cross-validated logistic regression (0.694). The convergence of models supported inclusion of four key predictors: age, physical job demands, musculoskeletal symptoms and mental health.

The optimal model demonstrated good calibration across risk deciles (Hosmer-Lemeshow χ^2^=5.40, *P* = 0.249) and minimal overfitting (bootstrap-corrected C-statistic 0.705), indicating stable performance for risk stratification applications.

### Proof of principle risk stratification tool

**Figure 2.**
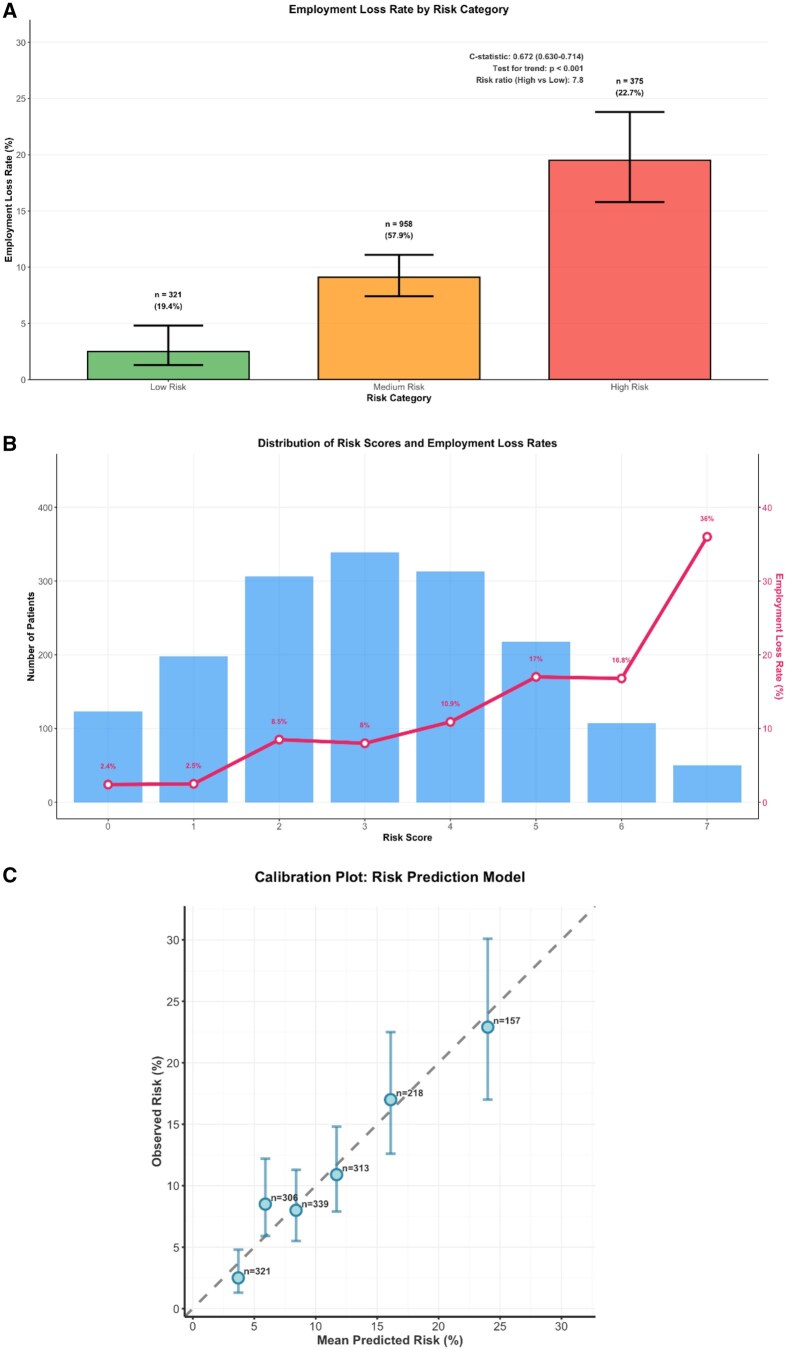
Development, performance and validation of the pragmatic work loss risk stratification tool. (A) Employment loss rates by three-category risk stratification with 95% CIs. (B) Patient distribution and employment loss rates across individual risk scores (0–7). (C) Calibration plot showing predicted versus observed work loss probabilities with perfect calibration line (dashed). The tool demonstrates good discrimination (C-statistic=0.672), significant risk stratification (*P* < 0.001) and excellent calibration (*P*=0.249)

An 8-point risk score derived from the optimal multivariable model stratified patients into three distinct risk categories with clinically meaningful differences in employment outcomes (Fig. 2 and Supplementary Table S2, available at *Rheumatology Advances in Practice* online):

- **Low risk (0–1 points)**: 321 patients (19.4%), 2.5% employment loss (95% CI: 1.3–4.8%)
- **Medium risk (2–4 points)**: 958 patients (57.8%), 9.1% employment loss (95% CI: 7.4–11.1%)
- **High risk (5–8 points)**: 375 patients (22.7%), 19.5% employment loss (95% CI: 15.8–23.8%)

The risk ratio for employment loss between high- and low-risk groups was 7.8 (95% CI: 3.9–15.6, *P* < 0.001), demonstrating strong discrimination across risk categories. When evaluated as a continuous predictor, the risk score maintained good discrimination (C-statistic 0.672) and calibration (Hosmer-Lemeshow *P* = 0.249), supporting its utility for clinical risk assessment.

### Robustness of findings

**Figure 3.**
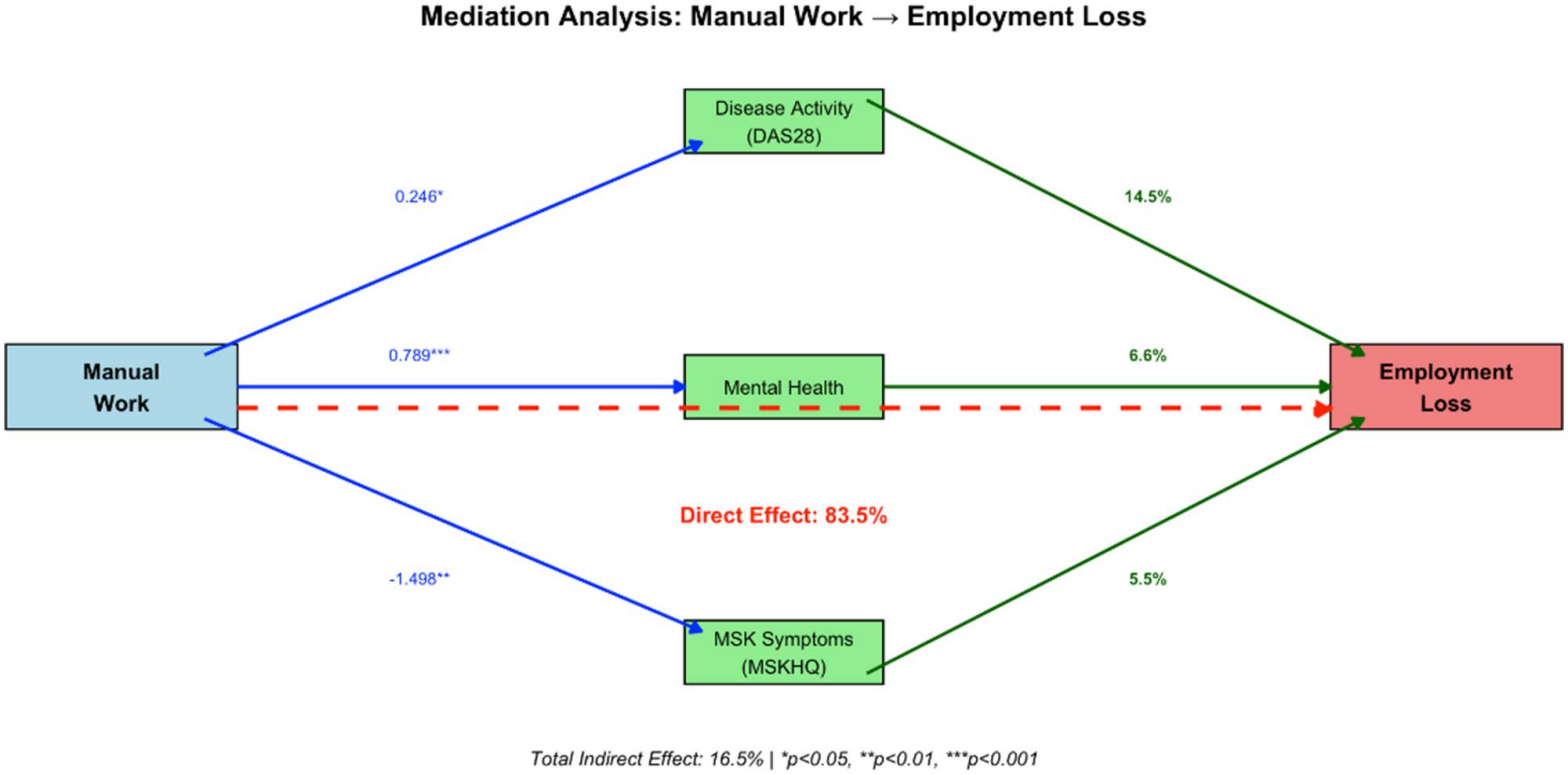
Mediation analysis of physically demanding occupation and work loss in early inflammatory arthritis. Blue arrows represent the pathways from manual work to each mediator, with coefficients showing the strength of association (0.246 for disease activity, 0.789 for mental health and −1.498 for musculoskeletal symptoms). Green arrows illustrate how each mediator contributes to employment loss, with percentages indicating the proportion of the total occupational effect mediated through each pathway: disease activity accounts for 14.5%, mental health for 6.6% and musculoskeletal symptoms for 5.5% of the association. The red dashed line represents the direct effect of manual work on employment loss that operates independently of these measured mediators, accounting for 83.5% of the total effect

A causal mediation analysis examined whether the occupational effect on employment loss operated through disease activity, mental health and musculoskeletal symptoms. Manual workers had significantly higher disease activity (coefficient 0.246, *P* = 0.001), worse mental health (coefficient 0.789, *P* < 0.001) and greater musculoskeletal impairment (coefficient −1.498, *P* = 0.009) (Fig. 3).

However, these clinical differences mediated only 16.5% of the total occupational effect on employment loss. The remaining 83.5% operated through direct pathways, indicating that employment loss primarily reflects structural job-body mismatch rather than inadequate disease control.

Of the 5287 patients working at baseline who had occupational data, 3625 (68.6%) lacked 3-month employment outcomes. Those with missing outcomes were younger than complete cases (mean age 49.4 vs 50.9 years, *P* < 0.001), less likely to be female (62.2% vs 67.1%, *P* < 0.001) and more likely to work in high physical demand occupations (20.2% vs 16.8%, *P* = 0.010). Disease activity did not differ significantly between groups (DAS28 4.53 vs 4.58, *P* = 0.147). Sensitivity analyses addressing substantial missing data (68.6%) demonstrated consistent associations between manual work and employment loss across multiple bias scenarios (IRR range 1.28–2.20), providing confidence in the robustness of findings (Supplementary Data S5, available at *Rheumatology Advances in Practice* online).

## Discussion

This national study of over 1600 working adults with early inflammatory arthritis had two complementary aims: first, to identify and quantify the key drivers of early employment loss; and second, to explore the feasibility of developing risk stratification tools using routinely available clinical data. Our explanatory analysis demonstrates how occupational physical demands, age, symptom burden and mental health are key determinants of employment loss within three months of diagnosis. Second, our prediction modelling work establishes proof-of-principle that a pragmatic risk stratification approach can identify an 8-fold gradient in employment risk (2.5–19.5%), potentially enabling targeted selection of patients for vocational rehabilitation interventions.

Our observation that patient-reported measures outperformed traditional disease activity assessments in predicting employment loss is congruent with other findings [12]. We have built on this evidence base by quantifying the role of occupational physical demands in early employment loss. Whilst the finding that manual workers face greater risk may seem intuitive, we have quantified this effect and shown that it operates largely independently of disease activity. Occupational physical demands showed stark effects, with more physically demanding roles experiencing 54% increased risk. Critically, mediation analysis revealed that only 16.5% of this occupational effect operated through disease activity, symptom burden and mental health. This unmediated effect exposes the harsh reality: workers in physical jobs face structural barriers that biologics cannot fix. Karasek’s demand-control model and the job demands-resources framework explain this through job strain, i.e. high demands with low control and inadequate resources for adaptation [13].

This finding aligns with the International Classification of Functioning, Disability and Health (ICF) framework, which conceptualizes work disability as arising from complex interactions between health conditions, personal factors and environmental contexts [14]. Within this biopsychosocial model, our identified predictors span the ICF domains: body functions (disease activity), activities (physical work demands), personal factors (age, mental health) and environmental factors (occupational context).

The impact on older workers deserves attention. Each 10-year increment in age increased risk of employment loss by 65%, with one in five workers aged 60 or older losing employment. Beyond the immediate 65% increased risk per decade, older workers face compressed timeframes for financial recovery, reduced re-employment prospects and potential pension losses. Unlike voluntary early retirement, arthritis-related job loss represents an involuntary transition with profound long-term consequences. Far from being less consequential near retirement, job loss in these final working years impacts pension entitlements, depletes retirement savings and leaves no time for financial recovery. For these workers, arthritis does not enable early retirement—it risks poverty.

We present the risk stratification tool as a proof-of-principle demonstration rather than a ready-for-implementation clinical tool. The moderate discrimination (C-statistic 0.71) and absence of external validation limit immediate application for individual patient decisions. However, the tool demonstrates sufficient performance for research applications, particularly stratifying participants in vocational rehabilitation studies or quality improvement initiatives. The 8-fold risk gradient provides meaningful stratification for population-level interventions, even if absolute differences may be insufficient for routine clinical decision-making.

Based on our risk categories, approximately 19% of working patients would be classified as low risk, 58% as moderate risk and 23% as high risk. Rather than guiding individual clinical decisions, this stratification could inform resource allocation, research recruitment and systematic approaches to occupational support referral.

The ‘Flags’ framework is a useful construct to illuminate the barriers to intervention to prevent job loss [15]. Whilst rheumatologists focus on ‘red flags’ of disease activity, our data suggest that ‘black flags’—workplace conditions and policies—may drive most employment loss. Solutions do not obviously lie in the control of a rheumatologist in the form of a tablet, injection or even a conversation in the clinic about work. The Sherbrooke model would suggest that multi-stakeholder interventions outperform clinic-based approaches, underscoring the futility of purely medical solutions [16, 17].

We acknowledge that external validation would be ideal, but datasets combining detailed occupational coding with work outcomes remain scarce. External validation could be pursued in future NEIAA cycles; the tool could be embedded within NEIAA data entry platforms or electronic health record templates to enable automated risk calculation at the point of care, supporting earlier identification of patients at risk of employment loss. For international audiences, the framework provides a template for local adaptation; the risk factors of age, occupation, mental health and symptom burden are universal. The tool’s strength lies in its pragmatism. Our approach uses data already collected, removing barriers to implementation. It aligns with EULAR’s 2021 points to consider, operationalizing their call for systematic approaches to work-oriented care and multi-stakeholder collaboration [8].

The economic argument is also relevant to discuss. Work disability costs society far more than healthcare, through lost productivity, benefit payments and family impacts. Early intervention using risk stratification could generate substantial returns through maintained employment, tax contributions and avoided welfare costs. In theory, NEIAA could embed work retention as a quality indicator; whilst this would help highlight the issues it would also be controversial given the locus of control to modify work retention does not lie in the hands of the rheumatologist.

Several limitations warrant consideration. First, the substantial missing outcome data (68.6%) raises concerns about selection bias. Our analysis of missing data patterns revealed that non-response was non-random, with younger patients, men and those in high physical demand occupations less likely to provide 3-month outcome data. This pattern is consistent with occupational health research where manual workers experiencing job loss may be harder to follow up due to healthcare disengagement, housing instability or job-seeking mobility. To address this, our sensitivity analyses under different missing-not-at-random assumptions that reflected plausible mechanisms for differential non-response. The consistent finding of increased employment loss risk for manual workers across all scenarios (IRR range 1.68–3.14) provides reassurance that our findings reflect genuine occupational vulnerabilities rather than an artefact of sampling bias.

Second, we intentionally focused on very early employment loss at 3 months. Although NEIAA collects work outcome data at both 3 and 12 months, the return rate for 12-month data was too low to allow robust analysis. We also did not examine treatment response as a predictor because disease-modifying therapies would have had limited time to exert measurable workplace benefits within this short interval. While longer-term outcomes would provide valuable insight into sustained work participation, job retention and the cumulative impact of disease, the early 3-month window is particularly informative. This is the point at which the initial impact of inflammatory arthritis on work ability is most pronounced, when symptoms are typically at their worst and when early support or workplace adjustments are most likely to influence longer-term employment trajectories. Nevertheless, focusing on such an early timeframe means that later employment losses, which may emerge as disease burden accumulates or as treatment response varies over time, are not captured. Future work using cohorts with extended follow-up would allow a more complete assessment of work outcomes over time.

Third, we need to acknowledge that within our MSKHQ score, item 6 assesses work difficulties in the preceding fortnight, potentially introducing circularity when predicting employment loss. We maintained the validated composite score for pragmatic reasons, though future research could explore the predictive value of individual MSK-HQ subsections. Finally, the tool’s moderate discrimination must be acknowledged, as it reflects the complex, multifactorial nature of work disability that extends beyond individual patient characteristics.

Future priorities include longitudinal validation, incorporation of workplace factors and development of digital tools facilitating patient-employer communication [18]. Implementation studies must test whether risk-stratified pathways deliver cost-effective outcomes. However, we cannot wait for perfect evidence while people lose their livelihoods.

Our tool demonstrates that four simple factors that we are required to record as part of a nationally mandated audit programme—age, job demands, symptom burden and mental health—effectively identify those facing imminent employment loss. However, when considering interventions, these findings demonstrate the predominance of direct occupational effects over disease-specific factors, highlighting the importance of workplace modifications and reasonable adjustments. For UK rheumatologists, the path forward is straightforward: we must normalize occupational risk assessment at diagnosis and establish clear referral pathways to vocational rehabilitation.

## Supplementary Material

rkaf149_Supplementary_Data

## Data Availability

Data access requests can be made through the Healthcare Quality Improvement Partnership.
