# Correction to: Development of a clinical tool to identify patients with early inflammatory arthritis at high risk of employment loss: analysis from the National Early Inflammatory Arthritis Audit

**DOI:** 10.1093/rap/rkag095

**Published:** 2026-08-11

This is a correction to: Ed Alveyn, Katie Bechman, Maryam Adas, Paul Amlani-Hatcher, Mrinalini Dey, Sarah Gallagher, Mark Gibson, Bethan Jones, Daksh Mehta, Sam Norton, Elizabeth Price, Mark Russell, Karen Walker-Bone, Elizabeth MacPhie, James Galloway, Development of a clinical tool to identify patients with early inflammatory arthritis at high risk of employment loss: analysis from the National Early Inflammatory Arthritis Audit, *Rheumatology Advances in Practice*, Volume 10, Issue 1, 2026, rkaf149, https://doi.org/10.1093/rap/rkaf149

In the originally published version of this manuscript, there was an error in the fifth co-author’s name. This has been corrected in the author list and ‘Author Contributions’ section to read “Mrinalini Dey”.

