# Supplementary material for: Development of a clinical tool to identify patients with early inflammatory arthritis at high risk of employment loss: analysis from the National Early Inflammatory Arthritis Audit
Source: Rheumatol Adv Pract. 2025 Dec 23;10(1):rkaf149. doi: 10.1093/rap/rkaf149 (PMC12798532; doi:10.1093/rap/rkaf149)
Supplement: rkaf149_Supplementary_Data [file rkaf149_supplementary_data.docx]

Supplementary

[**1. Detailed Methodology** 2](#_Toc214872745)

[**2. Proof-of-Principle Risk Stratification Model Development and Validation** 4](#_Toc214872746)

[**3. Pragmatic Risk Stratification Tool** 6](#_Toc214872747)

[**4. Mediation Analysis** 8](#_Toc214872748)

[**5. Missing Data Patterns** 9](#_Toc214872749)

# **1. Detailed Methodology**

We began from the hypothesis that patients working in occupations with greater physical demands would be at increased risk of early work loss following diagnosis of inflammatory arthritis. We further proposed that this effect would be mediated, at least in part, by disease activity and mental health burden, and that the influence of occupational demands might be modified by socioeconomic deprivation.

**Occupational classification**

Occupational classification was central to the study. Occupational and industry information was collected at baseline during routine clinical assessments using free-text fields in which patients were asked to provide both their job title and a brief description of their main activities. This generated rich but heterogeneous data, which required structured processing. We adopted a two-stage classification system. First, we developed a rule-based algorithm using regular expressions to assign Standard Occupational Classification (SOC) 2020 codes. The system matched common job titles and industry descriptors using hierarchical rules, prioritising specific matches over more general ones. This automated process successfully classified approximately 85% of responses, with full audit trails for transparency. Remaining cases were reviewed manually in batches. Two researchers independently classified ambiguous responses, resolving discrepancies by consensus. Responses indicating non-employment (such as “retired,” “student,” or “unemployed”) were excluded. Quality checks were undertaken on random samples from both automated and manual coding.

Several approaches to grouping occupations were explored. Options included a three-level classification of physical demands (low, moderate, high), groupings based on potential for remote or flexible work, fine-grained physical requirement categories, and industry-based groupings. Ultimately, we chose a binary classification of manual versus non-manual work. This choice was informed by clinical relevance—distinguishing work that requires significant physical exertion from primarily cognitive or administrative roles is particularly pertinent in musculoskeletal disease. Simplicity was also critical: any tool for clinical use must be practical in routine rheumatology practice. The binary classification maximised statistical power and aligned with conventions in the existing literature. Manual work included SOC groups 3, 5, 6, 7, 8, and 9 (associate professionals, skilled trades, service, sales, machine operatives, and elementary occupations), while non-manual work included SOC groups 1, 2, and 4 (managers, professionals, and administrative roles).

**Clinical and demographic variables**

Clinical and demographic variables were defined with similar care. Age was categorised into <50, 50–59, and ≥60 years, deliberately separating early retirement transitions from disease-related work loss. Disease activity was assessed using DAS28-CRP, with high activity defined as >5.1, reflecting thresholds for treatment escalation. Mental health burden was captured using a combined score from PHQ-2 and GAD-2 instruments, with established thresholds applied to identify probable anxiety or depression. Musculoskeletal impact was assessed with the MSKHQ, a validated 14-item patient-reported measure of function, pain, and participation. Scores ≤25 indicated significant impairment; this binary cut-point was chosen on the basis of score distribution, predictive value for employment outcomes, and simplicity for use in clinical practice.

**
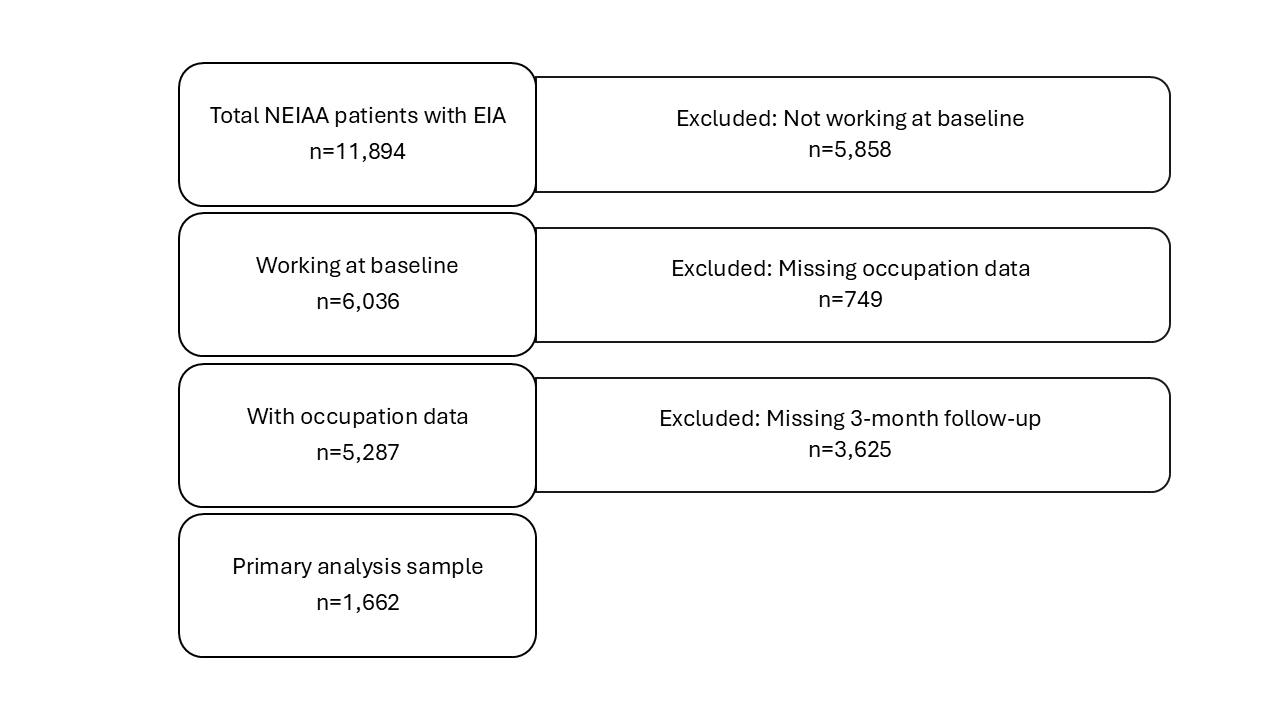
**

**Supplementary Figure S1. Study Flow Diagram.** *Flow diagram showing the selection of the primary analysis sample from the National Early Inflammatory Arthritis Audit (NEIAA).*

*Alternative text: A flow diagram shows the derivation of the primary analysis sample. The cohort begins with 11,894 NEIAA patients with early inflammatory arthritis. Of these, 5,858 are excluded because they were not working at baseline, leaving 6,036 working participants. A further 749 are excluded due to missing occupation data, leaving 5,287 with occupation information. Of these, 3,625 are excluded because they lack 3-month follow-up data. The final primary analysis sample includes 1,662 participants.*

# **2. Proof-of-Principle Risk Stratification Model Development and Validation**

Model development proceeded in a structured way. Penalised regression methods (LASSO and elastic net with 10-fold cross-validation) were applied to a comprehensive set of candidate variables, including physical job demands, age, sex, baseline disease activity, musculoskeletal health, mental health, socioeconomic deprivation, diagnosis type, and detailed occupational categories. This approach identified variables that contributed most to prediction when all information was considered simultaneously. Although using continuous DAS28 and MSK-HQ scores improved model performance, we made an *a priori* decision to dichotomise these measures, prioritising clinical simplicity and ease of interpretation over incremental gains in model accuracy. This approach enhances practicality for routine clinical use, where clear thresholds are often preferred.

Seven variables were initially selected: physical job demands, age, sex, baseline disease activity, musculoskeletal health, mental health, and diagnosis type. Both LASSO (λ = 0.0036) and elastic net (α = 0.5, λ = 0.0060) identified the same variables, excluding socioeconomic deprivation and detailed occupational categories. Diagnosis type contributed no predictive value when formally tested (Wald test: χ² = 0.28, p = 0.871) and was excluded from the final model.

Traditional regression models were then evaluated using the variables identified through penalised selection. Four candidate prediction models (standard logistic regression, LASSO elastic net and cross-validated logistic regression) were tested with sequential addition of predictor domains.

Standard logistic regression achieved optimal discrimination (C-statistic 0.708), outperforming LASSO (0.704), elastic net (0.704), and cross-validated logistic regression (0.694).

The optimal model, balancing predictive performance with clinical feasibility, combined physical job demands, age, musculoskeletal health, and mental health. Importantly, this model avoided reliance on clinician-recorded DAS28, enhancing its applicability in real-world practice.

Discrimination was assessed using the C-statistic. Calibration was evaluated with the Hosmer-Lemeshow goodness-of-fit test and calibration plots comparing predicted with observed risks across deciles. The optimal model demonstrated good calibration (Hosmer-Lemeshow χ² = 5.40, p = 0.249). Internal validation with 200 bootstrap iterations indicated minimal overfitting (bootstrap-corrected C-statistic 0.705) and provided bias-corrected performance estimates. Ten-fold cross-validation offered an additional estimate of performance on unseen data.

**Supplementary Table S2: Sequential Model Building - C-statistic Comparison**

| **Model** | **Variables** | **C-statistic** | **95% CI** | **N** |
| --- | --- | --- | --- | --- |
| **Model 1** | Age + Physical demands | 0.687 | 0.642-0.732 | 1,662 |
| **Model 2** | Model 1 + DAS28 high | 0.684 | 0.638-0.730 | 1,568 |
| **Model 3** | Model 2 + MSKHQ binary | 0.704 | 0.660-0.749 | 1,567 |
| **Model 4** | Model 1 + MSKHQ binary (no DAS28) | 0.706 | 0.663-0.749 | 1,661 |
| **Model 5** | Model 2 + Mental health | 0.696 | 0.650-0.742 | 1,561 |
| **Model 6** | Model 4 + Mental health | **0.710** | **0.667-0.753** | **1,654** |
| **Model 7** | Full model (all variables) | 0.708 | 0.663-0.752 | 1,560 |

**Legend:** C-statistic (area under ROC curve) with 95% confidence intervals for discrimination assessment. Model 6 selected as optimal based on highest C-statistic while maintaining clinical feasibility and near-maximal sample size. Physical demands were classified as manual versus non-manual work. MSKHQ binary defined as ≤25 versus >25. Mental health represents probable anxiety or depression, based on validated screening. Sample sizes vary due to missing covariate data.

**Model 6 Performance Details:**

- Bootstrap-corrected C-statistic: 0.705 (200 iterations)
- Hosmer-Lemeshow test: χ² = 5.40, p = 0.249 (good calibration)
- Brier score: 0.085

DAS28 = Disease Activity Score in 28 joints; MSKHQ = MSK Health Questionnaire.

# **3. Pragmatic Risk Stratification Tool**

From the optimal regression model we derived a risk tool to facilitate clinical application. Coefficients were converted into integer points proportional to their effect sizes: two points were assigned for manual work, severe musculoskeletal impairment, or age ≥60, and one point for moderate impairment, age 50–59, or clinically significant mental health burden. The lower weighting for mental health relates to the smaller effect size in the main Poisson model. Risk categories were defined by score distribution and clinical interpretability: low (0–1 points), medium (2–4), and high (5–8). Employment loss rates rose steeply across categories, from 2.5% in the low-risk group to 19.5% in the high-risk group, representing a nearly eightfold increase in risk.

Risk tool performance was evaluated using both discrimination and calibration metrics. The C-statistic was 0.672 (95% CI 0.632–0.712), indicating good discrimination. There was strong evidence of a trend across risk strata (p < 0.001). Calibration was good, with predicted and observed risks closely aligned; the Hosmer–Lemeshow test supported adequate fit (p = 0.249).

All analyses were conducted using Stata version 19 and R 4.3.0, with standard commands and packages for regression modelling, missing data analysis, and visualisation. Analyses followed TRIPOD guidelines throughout.

**Supplementary Table S3: Pragmatic Risk Stratification Tool for Employment Loss**

**Risk Score Components (0-8 points total)**

| **Component** | **Category** | **Points** |
| --- | --- | --- |
| **Physical job demands** | Non-manual (low demands) | 0 |
|  | Manual (moderate/high demands) | 2 |
| **Age** | <50 years | 0 |
|  | 50-60 years | 1 |
|  | >60 years | 2 |
| **MSK symptoms (MSKHQ)** | >30 (low impairment) | 0 |
|  | 21-30 (moderate impairment) | 1 |
|  | ≤20 (high impairment) | 2 |
| **Mental health** | No anxiety or depression | 0 |
|  | Probable anxiety or depression | 1 |

**Risk Score Categories and Outcomes**

| **Risk Category** | **Score Range** | **N (%)** | **Employment Loss** | **95% CI** | **Risk Ratio** |
| --- | --- | --- | --- | --- | --- |
| **Low Risk** | 0-1 points | 321 (19.4) | 8 (2.5%) | 1.3-4.8% | 1.0 (ref) |
| **Medium Risk** | 2-4 points | 958 (57.8) | 87 (9.1%) | 7.4-11.1% | 3.6 |
| **High Risk** | 5-8 points | 375 (22.7) | 73 (19.5%) | 15.8-23.8% | 7.8 |

**Tool Performance**

| **Metric** | **Value** |
| --- | --- |
| **C-statistic** | 0.672 (0.632-0.712) |
| **Test for trend** | p < 0.001 |
| **Calibration (Hosmer-Lemeshow)** | p = 0.249 |

**Legend:** Risk score derived from optimal multivariable model (Model 6) using coefficient-based point allocation. Manual work includes SOC groups 3,5,6,7,8,9. Non-manual work includes SOC groups 1,2,4. MSKHQ = MSK Health Questionnaire. Probable anxiety or depression based on validated PHQ-2/GAD-2 composite score. Employment loss defined as transition from employed at baseline to not employed at 3-month follow-up. Risk ratios calculated relative to low-risk group.

# **4. Mediation Analysis**

To examine potential causal pathways linking occupational demands to employment outcomes, we conducted mediation analysis using the ‘mediate’ command in Stata. This approach decomposes the total effect of manual work on employment loss into natural direct effects (operating independently of measured mediators) and natural indirect effects (operating through specific mediators).

We tested three candidate mediators separately: DAS28, mental health burden, and musculoskeletal symptom severity. Each mediation model specified a Poisson outcome model for employment loss and a linear mediator model, both adjusted for age and gender. The treatment variable was binary occupational classification (manual vs non-manual work). We used robust variance estimation and specified no treatment-mediator interaction.

Individual Mediator Effects

- DAS28: Among 1,568 patients with complete DAS28 data, the total effect of manual work on employment loss was 0.049 (95% CI 0.017–0.081, p=0.003). Disease activity mediated 0.004 (95% CI 0.000–0.008, p=0.053) of this effect, representing 7.9% of the total association. The natural direct effect remained substantial at 0.045 (95% CI 0.013–0.076, p=0.005).
- Mental Health: Among 1,655 patients with mental health data, the total effect was 0.054 (95% CI 0.022–0.085, p=0.001). Mental health symptoms mediated 0.007 (95% CI 0.002–0.011, p=0.004) of the occupational effect, accounting for 12.4% of the total association. The direct effect was 0.047 (95% CI 0.017–0.077, p=0.002).
- Musculoskeletal Symptoms: Among 1,661 patients with MSKHQ data, the total effect was 0.053 (95% CI 0.022–0.085, p=0.001). Musculoskeletal symptom burden mediated 0.005 (95% CI 0.000–0.009, p=0.037), representing 8.9% of the total effect. The direct effect remained large at 0.049 (95% CI 0.018–0.079, p=0.002).

Individually, the three tested mediators accounted for modest proportions of the occupational effect: disease activity (7.9%), mental health (12.4%), and musculoskeletal symptoms (8.9%). Collectively, these clinical and symptom-based pathways explain approximately 28% of the association between manual work and employment loss. The substantial remaining direct effect (approximately 72%) indicates that employment loss in manual workers operates predominantly through unmeasured occupational factors - such as physical job requirements, workplace inflexibility, or employer attitudes - rather than inadequate clinical disease control.

# **5. Missing Data Patterns**

Missing data were handled systematically. Of 6,036 patients in work at baseline, 5,287 (87.6%) had classifiable occupations. However, follow-up data at three months were available for only 1,662 (31.4%) individuals. This degree of attrition required a detailed assessment of potential bias.

**Supplemental Table S5. Comparison of Baseline Characteristics Between Patients with Complete and Missing 3-Month Employment Outcome Data**

| **Characteristic** | **Complete 3-month data (n=1,895)** | **Missing 3-month data (n=4,141)** | **P-value** |
| --- | --- | --- | --- |
| **Demographics** |  |  |  |
| Age, years, mean (SD) | 50.9 (11.8) | 49.4 (12.8) | <0.001 |
| Female, n (%) | 1,267 (66.9%) | 2,575 (62.2%) | <0.001 |
| **Physical demands of occupation** |  |  |  |
| Low physical demands, n (%) | 1,031 (54.4%) | 2,114 (51.0%) | 0.010 |
| Moderate physical demands, n (%) | 351 (18.5%) | 780 (18.8%) |  |
| High physical demands, n (%) | 280 (14.8%) | 731 (17.7%) |  |
| Missing/unclassified, n (%) | 233 (12.3%) | 516 (12.5%) |  |
| **Clinical characteristics** |  |  |  |
| DAS28, mean (SD) | 4.58 (1.35) | 4.53 (1.46) | 0.147 |

*Legend: Comparison of baseline characteristics between working patients at baseline who had complete 3-month employment outcome data versus those with missing outcome data. P-values from t-tests for continuous variables and chi-square tests for categorical variables. DAS28, Disease Activity Score in 28 joints; SD, standard deviation.*

Comparisons between complete and incomplete cases revealed several systematic differences. Patients with missing outcome data were slightly younger on average (49.4 vs 50.9 years, *p*<0.001), and women were more likely to have complete follow-up (67.1% vs 62.2%, *p*<0.001). Ethnic distribution also differed significantly between groups. By contrast, baseline disease activity, diagnosis type, and symptom duration did not differ between those with and without complete data. Occupational and socioeconomic patterns were evident: individuals in physically demanding roles were less likely to provide follow-up information, with response rates declining from 32.8% in low-demand roles to 27.7% in high-demand work. Follow-up was also less frequent among patients from more deprived areas, and there was marked regional variation across NHS regions.

**Inverse Probability Weighting**

To account for this non-random missingness, we developed a logistic regression model predicting the probability of complete follow-up, incorporating demographic, clinical, socioeconomic, and regional variables. Older age and female sex were associated with higher response rates, while Asian ethnicity was associated with lower response. Regional differences were also observed, with London and the North East showing the highest levels of follow-up. The model had modest explanatory power (pseudo R² = 0.0377), consistent with the multifactorial nature of follow-up patterns.

Weights were stabilised to prevent undue influence from outliers, yielding an effective sample size of 3,700. Weighted analyses confirmed the robustness of our findings, with manual work consistently associated with increased risk of early work loss.

**Sensitivity Analyses Under NMAR Assumptions**

We conducted four systematic sensitivity analyses under different missing-not-at-random (NMAR) assumptions, each reflecting plausible mechanisms for differential non-response patterns observed in occupational health research.

**Scenario 1:**

Differential Response Bias by Employment Outcome This scenario assumed that manual workers who lost employment were systematically less likely to provide follow-up data, reflecting potential embarrassment, housing instability, or healthcare disengagement following job loss. We modelled this by assigning imputed employment loss probabilities of 7.6% for non-manual work, 10.0% for moderate physical demand work, and 20.0% for high physical demand work among missing cases. Under these assumptions, manual work remained significantly associated with employment loss (RR 1.75, 95% CI 1.48-2.06, p<0.001).

**Scenario 2:**

Healthy Worker Effect This conservative scenario assumed that non-response predominantly reflected continued successful employment, with workers avoiding follow-up surveys when their condition had minimal impact. Imputed employment loss rates were set at 4.0% for non-manual work, 6.0% for moderate demand work, and 10.0% for high demand occupations. Even under these conservative assumptions favouring null findings, manual work effects persisted with strong significance (RR 1.73, 95% CI 1.41-2.13, p<0.001).

**Scenario 3:**

Extreme Occupational Selection To test robustness under maximum plausible bias, this scenario assigned extreme employment loss differentials: 5.0% for non-manual work, 15.0% for moderate demand work, and 30.0% for high demand occupations among missing cases. Rather than attenuating the occupational effect, this amplified associations, yielding an RR of 3.14 (95% CI 2.63-3.74, p<0.001) for manual work.

**Scenario 4:**

Informed Imputation with Systematic Underestimation This scenario used baseline clinical and demographic characteristics to predict employment outcomes among missing cases, subsequently applying a 20% inflation factor to account for potential systematic underestimation of employment loss in survey non-responders. A logistic regression model incorporating occupational demands, age, gender, disease activity, socioeconomic status, mental health, musculoskeletal symptoms, and work impairment was fitted among complete cases to generate predicted probabilities. Results remained robust with manual work showing RR 1.68 (95% CI 1.35-2.08, p<0.001).

Across all four NMAR scenarios, manual work was consistently associated with significantly increased employment loss risk, with RRs ranging from 1.68 to 3.14. Age effects remained consistently strong across all scenarios. The robustness of associations across divergent assumptions about missing data mechanisms provides confidence that our primary findings reflect genuine occupational vulnerabilities rather than methodological artefacts.
